# GATF-PCQA: A Graph Attention Transformer Fusion Network for Point Cloud Quality Assessment

**DOI:** 10.3390/jimaging11110387

**Published:** 2025-11-01

**Authors:** Abdelouahed Laazoufi, Mohammed El Hassouni, Hocine Cherifi

**Affiliations:** 1 Research Laboratory in Computer Science and Telecommunications (LRIT), Faculty of Sciences, Mohammed V University in Rabat, Rabat 1014, Morocco; 2 Faculty of Letters and Human Sciences in Rabat, Mohammed V University in Rabat, Rabat 8007, Morocco; mohamed.elhassouni@flsh.um5.ac.ma; 3 Carnot Interdisciplinary Laboratory of Burgundy (ICB) UMR 6303 CNRS, University of Burgundy, 21000 Dijon, France

**Keywords:** point cloud segmentation, no-reference quality assessment, graph neural networks, perceptual features, deep learning

## Abstract

Point cloud quality assessment remains a critical challenge due to the high dimensionality and irregular structure of 3D data, as well as the need to align objective predictions with human perception. To solve this, we suggest a novel graph-based learning architecture that integrates perceptual features with advanced graph neural networks. Our method consists of four main stages: First, key perceptual features, including curvature, saliency, and color, are extracted to capture relevant geometric and visual distortions. Second, a graph-based representation of the point cloud is created using these characteristics, where nodes represent perceptual clusters and weighted edges encode their feature similarities, yielding a structured adjacency matrix. Third, a novel Graph Attention Network Transformer Fusion (GATF) module dynamically refines the importance of these features and generates a unified, view-specific representation. Finally, a Graph Convolutional Network (GCN) regresses the fused features into a final quality score. We validate our approach on three benchmark datasets: ICIP2020, WPC, and SJTU-PCQA. Experimental results demonstrate that our method achieves high correlation with human subjective scores, outperforming existing state-of-the-art metrics by effectively modeling the perceptual mechanisms of quality judgment.

## 1. Introduction

The use of 3D models has surged in fields such as autonomous driving, robotics, remote sensing, and industrial inspection. However, processing techniques like compression and simplification often introduce distortions that degrade the visual quality of 3D point clouds. This has heightened demand for reliable ways to evaluate perceptual quality. Historically, human observers assessed distortion in 3D models—an approach that is laborious and costly. To increase efficiency, objective automated methods have been developed that emulate the judgments of an ideal human evaluator [1]. These metrics fall into three categories: full reference (FR) [2,3,4,5,6], reduced reference (RR) [7], and no reference (NR) [8,9,10,11,12,13,14]. Blind (NR) methods, which require a no-reference model, are becoming especially important in practical applications [15,16,17,18]. A 3D point cloud (PC) is defined by a set of points with geometric coordinates and, in many cases, additional attributes such as color, reflectance, or surface normals. Unlike images or videos, point clouds store irregularly distributed data representing object shape and geometry. This irregularity makes efficient feature extraction essential for reliable quality evaluation. Currently, very few NR-PCQA techniques have been developed. ResSCNN [11] applies voxelization to convert irregular points into a structured 3D grid. This representation enables the use of 3D convolutions to effectively aggregate local neighborhood information and predict quality scores. Chetouani et al. [19] propose a hybrid approach that extracts handcrafted descriptors from local patches and combines them with a CNN-based regression model. This design merges explicit geometric knowledge with the learning capacity of neural networks. Another common direction uses 2D representations, as in PQA-net [3] and IT-PCQA [20], which employ multi-view projections to convert 3D models into multiple rendered images for feature extraction. This allows them to utilize well-established image quality assessment features by treating the problem in a more mature domain. In contrast, Zhang et al. [12] employ a direct analysis of the point cloud’s attributes by modeling the statistical distributions of its geometry and color. Their method operates on the premise that visual distortions will manifest as measurable deviations in these underlying distributions. Beyond these, alternative paradigms have emerged, including video-based analysis [21] for assessing temporal quality in dynamic point clouds and domain adaptation strategies [20] that aim to transfer quality knowledge from labeled natural images to unlabeled point cloud views to overcome data scarcity. Recent advances have introduced more sophisticated frameworks, such as the work by Liu et al. [22], who propose D3-PCQA, a framework that bridges perceptual and quality domains—it achieves this through a domain-relevance degradation description designed to more accurately model the human visual system’s perception of various distortions—while Goswami et al. [23] develop PB-PCQA, a projection-based method that simulates human visual exploration. It generates multi-view 2D projections and employs a fusion mechanism to weigh their importance for a more perceptually aligned assessment. To capture complex dependencies, Zhu et al. [24] present COPP-Net, a patch-based framework that integrates PointNet++ for local feature extraction. It further incorporates a Transformer-based module to perform correlation analysis between patches and model long-range interactions. Similarly, Wu et al. [25] introduce CMDC-PCQA, a cross-modal deep-coupling model that jointly learns geometric and semantic features. It processes both the raw point cloud and its projected images to ensure quality prediction is informed by complementary information from multiple modalities. Li et al. [26] propose 3DRMF, which leverages PointNet++ for robust geometric and color feature extraction. A key innovation is their Rotational Mamba Fusion module, which is designed to efficiently model long-range sequences of features for a more holistic understanding of global context in quality assessment.

The current state of point cloud quality assessment still faces several key limitations. Point-based methods often emphasize geometric attributes while neglecting color cues, producing incomplete perceptual evaluations for content where chromatic details affect quality. Feature-based approaches depend heavily on the performance of handcrafted or learned feature extractors, where biased representations reduce prediction reliability. Projection-based methods lose information during 2D rendering and remain sensitive to viewpoint selection, which weakens score consistency. Most no-reference metrics also lack invariance to rotation, translation, and scale, causing unstable objective scores under different spatial conditions, even though subjective assessments allow unrestricted viewing. In addition, convolutional architectures used in learning-based models have limited receptive fields and fail to capture long-range structural relationships, restricting their ability to model complex geometric and textural dependencies essential for accurate quality prediction [27].

Recently, graph-based representations have attracted considerable attention in point cloud processing [28,29,30], owing to their ability to effectively model high-dimensional visual data by capturing local neighborhood structures and their perceptual relevance. Wang et al. [31] suggested building dynamic graphs for point clouds by repeatedly updating vertex characteristics using EdgeConv. The dynamic adjustment of graph connections based on spatial correlations is a crucial component of their methodology, as it allows for adaptive neighborhood creation for a better capture of local geometric features. He et al. [32] build both local and global graphs within and across voxel grids to capture structural dependencies at multiple scales. They incorporate point attention to strengthen feature extraction and introduce a sparse-to-dense regression module to aggregate multi-level features efficiently. This combination enables fine-grained spatial reasoning while preserving global context, improving accuracy in quality prediction tasks. Ren et al. [33] introduce graph-aware attention to adaptively encode graphs and a proposal-aware fusion module to integrate spatial information for refined predictions. Their model learns contextual relations across nodes and dynamically adjusts the attention weights according to spatial importance. Graphs provide a flexible and interpretable framework for representing 3D point clouds, where the connectivity between vertices encodes meaningful geometric and perceptual relationships. This capability has proven highly effective across a range of computer vision tasks, including segmentation and classification [34]. In the context of quality assessment, El Hassouni et al. [35] employed graph feature learning with random forest regression to evaluate the perceptual quality of colored meshes, while Lazzoufi et al. [36] introduced PCW-Graph, a no-reference framework based on a Graph Attention Fusion Network. However, these graph-based models used basic attention mechanisms and independent feature processing. Unlike earlier feature-based approaches [13,14,16,17,18], they lacked integrated multi-modal fusion. To address these limitations, we propose a Graph Attention Transformer-based point cloud quality assessment (GATF-PCQA) framework. The core GATF module performs dynamic cross-modal feature integration and importance weighting, providing an end-to-end learnable system that jointly models geometric and visual attributes within a unified graph structure. Traditional point-based methods fail to capture perceptual cues such as color and saliency, while projection-based ones suffer from information loss and viewpoint dependency. GATF-PCQA constructs a perceptually consistent graph representation by integrating geometric and visual features into clustered nodes connected through weighted edges reflecting feature similarity. The GATF module adaptively refines feature importance to capture complex distortions across content types and densities. Integrated with a Graph Convolutional Network (GCN), the framework provides end-to-end quality prediction consistent with human perception, improving robustness to distortion variations and enhancing cross-dataset generalization.

The main contributions of this study can be summarized as follows:

- A unified graph-based learning framework for no-reference point cloud quality assessment, which integrates geometric and perceptual features into a structured graph representation that aligns more closely with human quality perception.
- A novel Graph Attention Network Transformer Fusion (GATF) module, designed to dynamically refine and fuse multi-feature representations, enabling the model to capture both local distortions and global perceptual dependencies.
- End-to-end quality prediction, achieved by combining the GATF module with a Graph Convolutional Network (GCN) to regress fused features into objective quality scores that strongly correlate with subjective judgments.
- Comprehensive evaluation on three benchmark datasets (WPC, SJTU-PCQA, ICIP2020), demonstrating that the proposed method consistently outperforms state-of-the-art metrics in correlation with human perceptual scores.

This paper is structured as follows. We review related work in Section 2. We detail the proposed methodology in Section 3. Experimental results and analysis are presented in Section 4 and Section 5, and we conclude in Section 6.

## 2. Related Work

PCQA methods are commonly divided into projection-based and point cloud-based approaches, depending on the data representation. We give an overview of these methods in this section.

**Projection-Based Methods:** They assess point cloud quality by rendering 3D data into 2D images, enabling the use of established image quality assessment (IQA) metrics such as SSIM [2], MS-SSIM [37], IW-SSIM [38], and VIFP [39]. Hua et al. [40] introduced a blind evaluation technique that projects point clouds onto planes to extract geometric, color, and joint properties. In [41], Freitas et al. developed a full-reference (FR) measure that combined geometry and texture features from projected maps. Zhou et al. [42] created a reduced-reference (RR) method based on content-aware saliency-projection.

The integration of deep learning has significantly advanced no-reference (NR) projection-based strategies. Tao et al. [43] proposed a multi-scale feature fusion network that adaptively weights patch-level quality scores within a graph structure. Similarly, Liu et al. [8] developed a multi-task framework utilizing a deep neural network (DNN) to predict scores and probability vectors from multi-view projections. Extending this direction, Tu et al. [44] introduced a two-stream CNN designed to jointly capture features from both geometry and texture projection maps, while Yang et al. [20] incorporated adversarial domain adaptation to transfer quality knowledge across domains. Xie et al. [45] further advanced NR-PCQA by introducing a projection-based, multi-modal learning framework with graph-based fusion. Other works focus on resource efficiency and richer perceptual modeling. Zhang et al. [46] developed GMS-3DQA, a grid mini-patch method for quality prediction with reduced computational overhead, and Zhang et al. [47] extended this to multi-modal PCQA (MM-PCQA) by retrieving complementary modalities from point clouds. Shan et al. [48] proposed a contrastive pre-training approach leveraging mixed-distortion projections and semantic-guided multi-view fusion to learn quality-aware representations (CoPA). Zhang et al. [49] tackled viewpoint sensitivity with OP-HPVS, a method supported by the first human-annotated viewpoint database and a novel human preference index. Finally, Shan et al. [50] proposed DisPA, which employs a dual-branch network with mutual information minimization to disentangle content and distortion cues.

**Point-based Methods:** They directly operate on raw point cloud data, extracting geometric, color, and other attributes for quality evaluation. Early work by Mekuria and Tian introduced the point-based metrics PSNR_p2po [51] and PSNR_p2pl [3], marking the foundation of PCQA research. This was later extended with PSNR_yuv [52] to assess texture distortions in colored point clouds. Alexiou et al. [53] measured degradation via angular differences between corresponding points, while Meynet et al. [54] proposed MSDM, relying on local curvature statistics. Javaheri et al. [55] further employed the generalized Hausdorff distance as a quality indicator. Beyond geometry, color information has been extensively explored. Meynet et al. [4] improved MSDM by integrating color features, and Viola et al. [56] quantified distortion using global color statistics. In feature space, Alexiou et al. [57] developed a structural similarity index by fusing color and geometric descriptors. In [58,59,60], Diniz et al. proposed methods based on local luminance patterns (LLP) and local binary patterns (LBP), while Yang et al. [5,61] introduced a quality prediction method that combines multi-scale potential energy differentials (MPED) with local graph representations.

Reduced-reference (RR) methods are particularly valuable when full-reference point clouds are unavailable. Viola et al. [7] introduced an RR metric using linear optimization to combine geometry, brightness, and normal-based features. Liu et al. predicted V-PCC compression quality via geometric and color features [62]. Su et al. employed Support Vector Regression (SVR) for RR assessment [63].

However, in practice, reference point clouds are not always available, prompting interest in no-reference (NR) approaches. The 3D-NSS-based model presented by Zhang et al. [12] regresses quality using SVR and extracts color and geometric features. Zhou et al. [64] suggested a structure-guided resampling strategy for blind quality evaluation. Shan et al. [65] developed NR-PCQA with PAME, a self-supervised pre-training framework built on masked autoencoders that use projected images to learn representations that are both content and distortion-aware.

Deep learning has further driven progress in PCQA. Chetouani et al. [19] applied deep neural networks (DNNs) to map feature embeddings to quality scores. Liu et al. [11] developed an NR-PCQA model using sparse CNNs, and the multi-task Graph Convolutional Network for NR-PCQA, GPA-Net, was presented by Shan et al. [66]. In [67], Wang et al. proposed a non-local graph framework that aggregates geometric and color gradients with multi-task learning. In [68], Tliba et al. employed a Dynamic Graph CNN, while Su et al. [69] and Liu et al. [70] explored bitstream-based neural networks for perceptual quality prediction, particularly for V-PCC encoded point clouds.

## 3. Proposed Method

**Figure 1:**
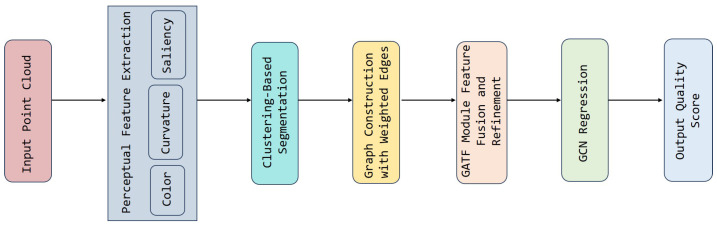
High-level workflow of the proposed GATF-PCQA framework, showing the main processing stages from input point cloud to quality score prediction.

**Figure 2:**
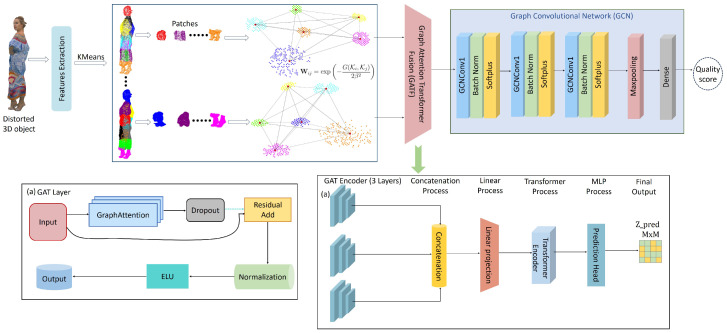
The general framework of the proposed method.

The overall pipeline of the proposed GATF-PCQA framework is illustrated in Figure 1, while the detailed architectural components are shown in Figure 2. The framework consists of four main stages designed to transform a raw point cloud into a perceptually aligned quality score. The process begins with perceptual feature extraction, where low-level curvature, saliency, and color characteristics are computed to capture the fundamental geometric and visual distortions that impact human perception. These features are then structurally organized in the graph-based representation phase; we construct a weighted graph where nodes are formed from perceptually significant clusters of points, and edges are weighted based on multi-feature similarity, effectively encoding the point cloud’s structural relationships into an adjacency matrix. This graph is subsequently processed by our novel Graph Attention Network Transformer Fusion (GATF) module, which intelligently refines the feature set by dynamically adjusting the importance of each feature through attention mechanisms and Transformer layers, yielding a robust, view-specific representation. Finally, in the quality prediction stage, a Graph Convolutional Network (GCN) performs regression on this refined representation to estimate a final quality score that accurately predicts human subjective judgments.

### 3.1. Extraction of Perceptual Features

Given a point cloud $\mathrm{PC}={\left\{p_{i}\right\}}_{i=1}^{N}$, we obtain the perceptual feature set $F_{\mathrm{perc}}$ as

(1) $$F_{\mathrm{perc}}=F_{\mathrm{perc}}\left(\mathrm{PC}\right),$$

where $F_{\mathrm{perc}}$ is the function used to extract perceptual features. The selected features, curvature, saliency, and color, were chosen for their complementary roles in capturing essential perceptual attributes relevant to evaluating the quality of point clouds. Color provides critical information for human visual perception, particularly in textured or chromatically rich scenes, enabling the detection of distortions such as color bleeding, desaturation, or compression artifacts that are imperceptible in geometry-only analyses. Curvature characterizes local geometric surface properties, emphasizing edges, corners, and fine details, thereby supporting the evaluation of geometric fidelity in the presence of noise, smoothing, or detail loss. Saliency identifies visually prominent regions that attract human attention, allowing the model to focus on perceptually important areas and improving alignment with subjective human judgments. Together, these features form a comprehensive and well-balanced perceptual representation that incorporates structural and visual information, leading to more reliable and human-consistent quality predictions. Table 1 shows a summary of perceptual features used in the proposed method.

### 3.2. Clustering-Based Point Cloud Segmentation

Clustering algorithms group data points according to similarity measures, operating without prior assumptions about the underlying data structure. Within the context of point cloud quality assessment, this process enables the identification of coherent regions sharing common geometric or perceptual attributes within the point cloud. This regional segmentation facilitates localized distortion analysis and models spatial dependencies, thereby enhancing the perceptual relevance of quality metrics through the capture of contextual inter-point relationships.

A variety of clustering approaches have been explored for point cloud processing, including Spectral Clustering [71], Mean Shift [72], Watershed [73], DBSCAN [74], KD-Tree [75], and K-means [76]. Each technique presents distinct advantages and limitations for PCQA:

- **Density-based methods (DBSCAN)** automatically determine cluster numbers but struggle with varying point densities and require careful parameter tuning.
- **Mean Shift** does not assume spherical clusters but is computationally intensive for large point clouds.
- **Spectral Clustering** effectively captures non-convex structures but has high computational complexity.
- **Watershed** is sensitive to noise and often leads to over-segmentation.

Among these, K-means [77,78] was selected for our framework due to several key advantages aligned with PCQA requirements: (1) computational efficiency for large-scale point clouds; (2) deterministic results ensuring reproducible quality scores; (3) effective capture of spherical clusters in perceptual feature space; and (4) straightforward integration with subsequent graph construction. The algorithm is applied to the perceptual feature set $F_{\mathrm{perc}}$ to segment the distorted point cloud into *K* clusters, which subsequently serve as nodes in the graph representation, forming the foundation for quality assessment.

### 3.3. Graph Construction with Perceptual Weights

A weighted graph G=(V,E,W) models the structural dependencies among perceptual clusters, with V representing cluster nodes, E defining the edges between them, and $W\in R^{K\times K}$ encoding edge weights based on geometric closeness and perceptual similarity. The creation of the graph transforms clustered perceptual features $F_{\mathrm{perc}}$ into a relational representation suitable for quality evaluation.

The node set $V=\{K_{1},\ldots ,K_{K}\}$ refers to the *K* perceptual clusters generated by K-means segmentation (Section 3.2), where each node $K_{i}$ aggregates points of similar perceptual properties. Edges (i,j)∈E are defined between clusters $K_{i}$ and $K_{j}$ when the Euclidean distance between their centroids is smaller than a predefined radius *r*. The weight matrix W encodes the interaction strength by integrating geometric distance and perceptual feature similarity.

(2) $$W_{ij}=\left\{\begin{array}{cc} \mathrm{Sim}\left(K_{i},K_{j}\right)\cdot G\left(K_{i},K_{j}\right) & \mathrm{if}\;K_{j}\in N_{r}\left(K_{i}\right)\\ 0 & \mathrm{Otherwise} \end{array}\right.$$

For every cluster $K_{i}$, we compute its perceptual centroid $\nu_{i}\in R^{d}$ as the average of the feature vectors of its member points:

(3) $$\nu_{i}=\frac{1}{|K_{i}|}\sum_{p_{m}\in K_{i}}F_{\mathrm{perc}}\left(p_{m}\right),$$

where $|K_{i}|$ is the number of points in cluster $K_{i}$ and $F_{\mathrm{perc}}\left(p_{m}\right)$ denotes the perceptual characteristics, curvature, saliency, and color of point $p_{m}$.

The Euclidean distance between cluster centroids represents geometric dissimilarity defined as

(4) $$G\left(K_{i},K_{j}\right)={∥\nu_{i}-\nu_{j}∥}_{2}=\sqrt{\sum_{d=1}^{3}{\left(\nu_{i}^{\left(d\right)}-\nu_{j}^{\left(d\right)}\right)}^{2}},$$

where $\nu_{i}^{\left(d\right)}$ corresponds to the *d*-th coordinate (X, Y, or Z) of centroid $\nu_{i}$.

Perceptual similarity is estimated through a radial basis function (RBF) kernel that maps distances to similarity measures:

(5) $$\mathrm{Sim}\left(K_{i},K_{j}\right)=\exp\left(-\frac{G(K_{i},K_{j})}{2\beta^{2}}\right),$$

with β>0 controlling the decay rate. Smaller β emphasizes local connections, while larger β allows broader interactions. Empirically, we set β=0.3r, where the neighborhood radius from Section 3.2 is denoted by *r*.

The edge weights are calculated by combining geometric proximity and perceptual similarity, ensuring that clusters which are both close in space and perceptually similar are strongly connected. The proposed dual-metric adjacency matrix retains essential structural and perceptual dependencies and reduces computational complexity by operating at the cluster level.

### 3.4. Graph Attention Mechanism

The Graph Attention Layer is a fundamental, single-head attention mechanism that drives the entire architecture. Our implementation builds upon the Graph Attention Network (GAT) framework [79]. The goal is to compute a new representation for each node as a weighted aggregation of the features of its neighboring nodes based on the graph’s edge index. The crucial innovation is that these weights are dynamic, computed through a self-attention mechanism that builds on the features of the source node as well as the target node. This enables the model to adaptively rank the neighborhood’s most informative connections, which is essential for identifying intricate, non-Euclidean interactions in graph data.

The initial transformation phase employs a linear projection operation defined as

(6) $$H=XW\;\mathrm{where}\;W\in R^{d_{\mathrm{in}}\times \left(d_{\mathrm{out}}\times h\right)}$$

where *H* represents the transformed feature matrix, $X\in R^{N\times d_{\mathrm{in}}}$ denotes the input feature matrix with *N* nodes and input dimension $d_{\mathrm{in}}$, *W* is the learnable weight matrix, $d_{\mathrm{out}}$ signifies the output feature dimension per attention head, and *h* indicates the number of parallel attention heads. The attention mechanism then computes source and target projections through

(7) $$H_{\mathrm{src}}=\mathrm{attn}\_\mathrm{src}\left(H\left[\mathrm{src}\right]\right)\;H_{\mathrm{dst}}=\mathrm{attn}\_\mathrm{dst}\left(H\left[\mathrm{dst}\right]\right)$$

where $H_{\mathrm{src}}$ and $H_{\mathrm{dst}}\in R^{\left|\mathrm{edges}\right|\times d_{\mathrm{out}}}$ represent the source and target node features after attention-specific linear transformations. Here, attn_src and attn_dst are distinct learnable linear projection layers (or multi-layer perceptrons) that transform the source and target node features into a shared latent space suitable for computing attention scores. The parameters src and dst denote the source and target node indices extracted from the edge connectivity information. The attention scoring mechanism proceeds through

(8) $$E_{ij}=\mathrm{LeakyReLU}\left(H_{\mathrm{src}}^{\left(i\right)}+H_{\mathrm{dst}}^{\left(j\right)}\right)$$

(9) $$A_{ij}=\sum_{k=1}^{d_{\mathrm{out}}}E_{ijk}\cdot W_{{\mathrm{attn}}_{k}}$$

where $E_{ij}$ constitutes the raw attention scores between nodes *i* and *j*, $A_{ij}$ represents the final attention scores, LeakyReLU denotes the leaky rectified linear unit activation function with negative slope parameter 0.2, and $W_{\mathrm{attn}}\in R^{d_{\mathrm{out}}}$ embodies the learnable attention weight vector. The normalized attention coefficients are obtained via softmax normalization:

(10) $$\alpha_{ij}=\frac{\exp(A_{ij})}{\sum_{k=1}^{N}\exp\left(A_{ik}\right)}$$

where $\alpha_{ij}\in \left[0,1\right]$ signifies the normalized attention coefficient quantifying the importance of node *j*’s features to node *i*, and exp represents the exponential function. The final aggregated features emerge through weighted summation:

(11) $$H_{\mathrm{out}}=\sum_{j=1}^{N}\alpha_{ij}H_{j}$$

where $H_{\mathrm{out}}\in R^{N\times (d_{\mathrm{out}}\times h)}$ constitutes the output feature matrix after multi-head attention aggregation, and $H_{j}$ denotes the features of node *j* from the transformed matrix *H*.

### 3.5. GAT Layer

The GAT Layer serves as a critical architectural component that integrates the foundational Graph Attention Layer into a stable and powerful neural network block. This module significantly extends the basic attention mechanism by incorporating multi-head attention, augmented with standard deep learning techniques to enhance performance and training stability. The multi-head mechanism enables the model to capture information from disparate representation subspaces concurrently, thereby substantially enriching its representational capacity. To ensure robust learning, several key elements are incorporated: dropout is applied as a regularization technique to mitigate overfitting; residual connections are employed to address the vanishing gradient problem, permitting the effective training of deeper architectures; and layer normalization is implemented to stabilize the learning process through feature-wise activation normalization. Consequently, the GAT Layer functions as a vital, deep-learning-ready construct that can be efficiently stacked to form sophisticated and high-performing graph encoder networks. The GAT Layer processing begins by computing the multi-head attention features:

(12) $$H_{\mathrm{attn}}=\mathrm{GraphAttentionLayer}\left(X,\mathrm{edge}\_\mathrm{index}\right)$$

(13) $$H_{\mathrm{drop}}=\mathrm{Dropout}\left(H_{\mathrm{attn}}\right)$$

(14) $$H_{\mathrm{res}}=\left\{\begin{array}{cc} H_{\mathrm{drop}}+X & \mathrm{if}\;d_{\mathrm{out}}\times h=d_{\mathrm{in}}\\ H_{\mathrm{drop}} & \mathrm{otherwise} \end{array}\right.$$

(15) $$H_{\mathrm{norm}}=\mathrm{LayerNorm}\left(H_{\mathrm{res}}\right)$$

(16) $$H_{\mathrm{out}}=\mathrm{ELU}\left(H_{\mathrm{norm}}\right)$$

where $H_{\mathrm{attn}}\in R^{N\times (d_{\mathrm{out}}\times h)}$ represents the multi-head attention output, $\mathrm{edge}\_\mathrm{index}\in R^{2\times |\mathrm{edges}|}$ contains the graph connectivity information, $H_{\mathrm{drop}}$ indicates the attention output after dropout regularization with probability 0.3, $H_{\mathrm{res}}$ incorporates residual connections when dimension matching occurs, $H_{\mathrm{norm}}$ signifies layer-normalized features, and ELU denotes the exponential linear unit activation function.

### 3.6. Fusion Transformer

The Fusion Transformer module is designed for the integrative synthesis of multi-modal feature embeddings (saliency, curvature, color) by modeling high-order interactions across their respective representation spaces. Unlike localized graph operations, it constructs a global fully connected graph from all modality-aligned nodes and employs self-attention to enable cross-modal node-to-node contextualization. This self-attention mechanism, popularized by the Transformer architecture [80], facilitates the discovery of latent, non-local dependencies that are inaccessible through isolated feature processing. The paramount importance of this module lies in its capacity to achieve genuine multi-modal fusion. It transcends elementary feature combination strategies, such as concatenation or averaging, by implementing a dynamic, attention-weighted synthesis of feature information. This learned integration is critical for the accurate reconstruction of the global adjacency matrix, a task that necessitates a comprehensive understanding of complex, cross-modal interactions. The fusion Transformer module processes concatenated multi-modal features through

(17) $$H_{\mathrm{proj}}=\mathrm{Linear}\left(H_{\mathrm{concat}}\right)\;\mathrm{where}\;H_{\mathrm{concat}}\in R^{N\times (3\times d_{\mathrm{hidden}})}$$

(18) $$H_{\mathrm{trans}}=\mathrm{TransformerEncoder}\left(H_{\mathrm{proj}}\right)$$

where $H_{\mathrm{proj}}\in R^{N\times d_{\mathrm{hidden}}}$ represents the projected feature matrix, $H_{\mathrm{concat}}$ contains concatenated features from three modalities (saliency, curvature, color), each of dimension $d_{\mathrm{hidden}}$, and $H_{\mathrm{trans}}$ embodies the Transformer-encoded features after processing through multiple encoder layers with four attention heads.

### 3.7. Graph Attention Network Transformer Fusion (GATF)

The Graph Attention Network Transformer Fusion (GATF) is a sophisticated multi-modal deep learning architecture that integrates three different feature modalities (saliency, curvature, and color features) using a hierarchical processing approach. The model combines the strengths of Graph Attention Networks (GATs) for processing graph-structured data with Transformer encoders for global context modeling and feature fusion. The architecture processes each feature modality through dedicated GAT encoder stacks, fuses the representations using a Transformer-based fusion module, and finally reconstructs the adjacency matrix through a prediction head with outer product operation.

The complete architectural flow culminates in adjacency matrix prediction through

(19) $$H_{\mathrm{sal}}^{\left(L\right)}={\mathrm{GAT}}_{\mathrm{sal}}^{\left(L\right)}\left(\cdots {\mathrm{GAT}}_{\mathrm{sal}}^{\left(1\right)}\left(X_{\mathrm{sal}}\right)\cdots \right)$$

(20) $$H_{\mathrm{curv}}^{\left(L\right)}={\mathrm{GAT}}_{\mathrm{curv}}^{\left(L\right)}\left(\cdots {\mathrm{GAT}}_{\mathrm{curv}}^{\left(1\right)}\left(X_{\mathrm{curv}}\right)\cdots \right)$$

(21) $$H_{\mathrm{col}}^{\left(L\right)}={\mathrm{GAT}}_{\mathrm{col}}^{\left(L\right)}\left(\cdots {\mathrm{GAT}}_{\mathrm{col}}^{\left(1\right)}\left(X_{\mathrm{col}}\right)\cdots \right)$$

(22) $$H_{\mathrm{fused}}=\mathrm{TransformerEncoder}\left(\mathrm{Linear}\left(\mathrm{Concat}\left(H_{\mathrm{sal}}^{\left(L\right)},H_{\mathrm{curv}}^{\left(L\right)},H_{\mathrm{col}}^{\left(L\right)}\right)\right)\right)$$

(23) $$P=\sigma (W_{2}\left(\mathrm{ELU}\left(W_{1}\left(\mathrm{ELU}\left(W_{0}H_{\mathrm{fused}}+b_{0}\right)\right)+b_{1}\right)\right)+b_{2})$$

(24) $$Z_{\mathrm{pred}}=PP^{T}$$

where $X_{\mathrm{sal}},X_{\mathrm{curv}},X_{\mathrm{col}}\in R^{N_{\mathrm{modality}}\times d_{\mathrm{in}}}$ represent input features for saliency, curvature, and color modalities, respectively, $H_{\mathrm{modality}}^{\left(L\right)}\in R^{N_{\mathrm{modality}}\times \left(d_{hidden,L}\times h\right)}$ denotes the final layer features for each modality, $H_{\mathrm{fused}}\in R^{N_{\max}\times d_{hidden,L}}$ constitutes the fused feature representation, $P\in R^{N_{\max}\times 1}$ represents node prediction probabilities after processing through multi-layer perceptron with weight matrices $W_{0}\in R^{d_{hidden,L}\times d_{hidden,L}}$, $W_{1}\in R^{d_{hidden,L}\times \frac{d_{hidden,L}}{2}}$, and $W_{2}\in R^{\frac{d_{hidden,L}}{2}\times 1}$ with corresponding bias terms $b_{0}$, $b_{1}$, and $b_{2}$, σ denotes the sigmoid activation function, and $A_{\mathrm{pred}}\in R^{N_{\max}\times N_{\max}}$ represents the final predicted adjacency matrix obtained through outer product operation $PP^{T}$.

### 3.8. Graph Convolutional Network (GCN)

The Graph Convolutional Network (GCN) serves as the final regressor in our PCQA pipeline, transforming the fused graph representation from the GATF module into a singular perceptual quality score. Its importance is to propagate and aggregate features throughout the graph structure, which allows the model to capture both global structural links and localized distortions that are important for evaluating quality. The GCN integrates contextual information from nearby nodes to hierarchically refine node properties through its message-passing mechanism. This graph-level processing is stabilized via batch normalization and non-linear Softplus activations. A final global max pooling layer generates a permutation-invariant graph embedding, which a dense layer regresses to the predicted Mean Opinion Score (MOS), ensuring a direct mapping between the point cloud’s graph topology and human perception.

The GCN uses the predicted adjacency matrix Zpred from the GATF module as input. The process begins with a graph convolutional layer, which refines the node representations by performing a normalized aggregation of features from adjacent nodes. For a given layer *l*, this operation is formally defined as

(25) $$H^{\left(l+1\right)}=\sigma \left({\hat{D}}^{-\frac{1}{2}}\hat{Z}\mathrm{pred}{\hat{D}}^{-\frac{1}{2}}H^{\left(l\right)}W^{\left(l\right)}\right)$$

where $H^{\left(l\right)}$ is the input node feature matrix at layer *l* (with the initial input $H^{\left(0\right)}=X\deg$, the matrix of node degrees calculated from Zpred), $\hat{Z}\mathrm{pred}=Z\mathrm{pred}+IN$ is the predicted adjacency matrix with added self-connections, $\hat{D}$ is its corresponding degree matrix where $\hat{D}ii=\sum_{j}\hat{Z}\mathrm{pred},ij$, $W^{\left(l\right)}$ is a layer-specific trainable weight matrix, and σ denotes the activation function. The output of this operation is subsequently passed through a batch normalization layer and a non-linear activation function to stabilize training and enhance representational capacity:

(26) $$Z_{\mathrm{norm}}^{\left(l+1\right)}=\mathrm{BN}\left(H^{\left(l+1\right)}\right)$$

(27) $${\tilde{H}}^{\left(l+1\right)}=\mathrm{Softplus}\left(Z_{\mathrm{norm}}^{\left(l+1\right)}\right)$$

where BN(·) denotes batch normalization and $\mathrm{Softplus}\left(x\right)=\log\left(1+e^{x}\right)$. This sequence of graph convolution, normalization, and activation is repeated through multiple layers. The resulting node features ${\tilde{H}}^{\left(L\right)}$ from the final layer *L* are then aggregated into a graph-level embedding vector using global max pooling:

(28) $$h_{G}=\mathrm{GlobalMaxPool}\left({\tilde{H}}^{\left(L\right)}\right)$$

where $h_{G}$ encapsulates the holistic quality information. Finally, this graph embedding is transformed into the predicted quality score via a linear regression layer:

(29) $$\hat{MOS}=w_{r}^{T}h_{G}+b_{r}$$

where $w_{r}$ and $b_{r}$ are learnable parameters, and $\hat{MOS}$ is the final output predicting human perceptual quality.

### 3.9. Quality Prediction

For quality prediction, the fused feature matrix $Z_{\mathrm{pred}}\in R^{k\times d_{\mathrm{fused}}}$, obtained by integrating the GATF-derived adjacency matrix A with perceptual features $F_{\mathrm{perc}}$, is processed by a Graph Convolutional Network (GCN). The GCN is applied to the cluster graph $G=(\left\{C_{i}\right\},Z_{\mathrm{pred}})$, in which each node represents a cluster defined in Section 3.3, and the edges capture the feature-driven dependencies obtained through fusion.

Our GCN architecture is composed of three graph convolutional layers with hidden dimensions of 64, 64, and 32, each followed by batch normalization and a Softplus activation to stabilize and enrich feature learning. A global max pooling layer is then applied to aggregate node-level features into a compact graph-level representation. Finally, a fully connected dense layer outputs the predicted quality score, enabling effective regression for point cloud quality assessment.

The network derives quality scores by progressively processing data through graph convolutional layers:

(30) $$Q_{\mathrm{pred}}=\mathrm{GCN}\left(Z_{\mathrm{pred}}\right)=\mathrm{MaxPool}\left(\overset{L}{\underset{\ell =1}{⨁}}Softplus\left(\hat{A}H^{\left(\ell -1\right)}W^{\left(\ell \right)}\right)\right),$$

where $H^{\left(0\right)}=Z_{\mathrm{pred}}$ denotes the fused input features matrix, *L* indicates the number of graph convolutional layers, $\hat{A}$ is the normalized adjacency matrix with self-loops, and $W^{\left(\ell \right)}$ are the trainable weights of the *ℓ*-th layer. The operator ⨁ represents layer-wise feature aggregation, and MaxPool(·) aggregates cluster-level embeddings into a global representation. The final scalar $Q_{\mathrm{pred}}\in R$ corresponds to the predicted perceptual quality score.

### 3.10. Graph Convolutional Network Training

**Figure 3:**
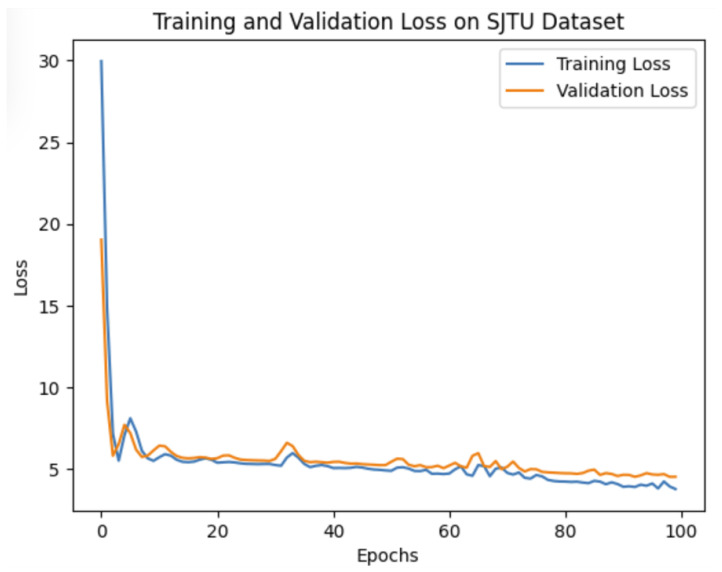
Training and validation loss curves on the SJTU-PCQA dataset.

**Figure 4:**
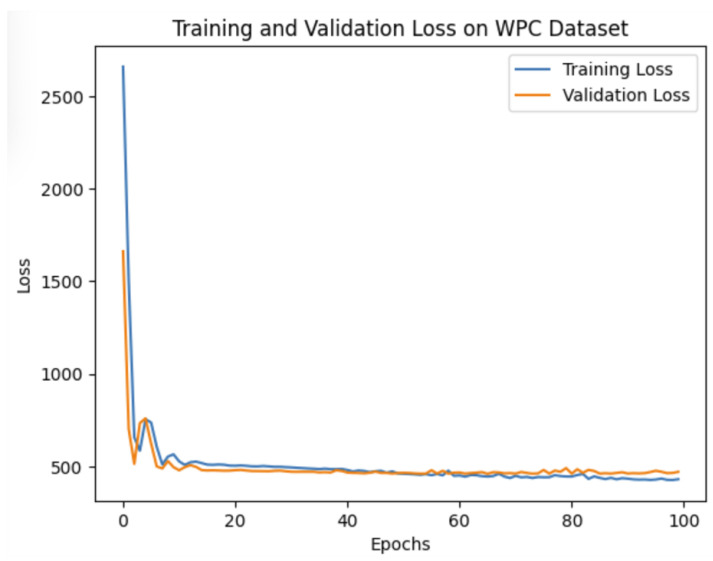
Training and validation loss curves on the WPC dataset.

Training is performed using backpropagation with the Adam optimizer, initialized at a learning rate of $1\times 10^{-4}$ and a batch size of 32. The dataset is partitioned into training (80%), validation (10%), and test (10%) subsets, ensuring disjoint reference point clouds across partitions. The objective function aims to minimize the error between the predicted quality scores $Q_{\mathrm{pred}}$ and the corresponding ground-truth Mean Opinion Scores (MOSs):

(31) $$L_{\mathrm{MSE}}=\frac{1}{\left|D\right|}\sum_{i=1}^{\left|D\right|}{\left(Q_{\mathrm{pred}}^{\left(i\right)}-{\mathrm{MOS}}^{\left(i\right)}\right)}^{2}$$

where |D| represents the number of distorted point clouds in the training set. Softplus activation functions are used to provide smooth non-linear transformations, non-negative, and gradient-friendly feature propagation, avoid dead neurons, and align naturally with distortion-related features in point clouds. It provides a good balance between expressivity (capturing subtle degradations) and stability (avoiding saturation/vanishing gradients). The model is trained for 100 epochs with early stopping determined by validation performance, preserving the configuration yielding the highest alignment between predicted and subjective scores. Figure 3 and Figure 4 present the training and validation loss curves on the SJTU-PCQA and WPC datasets. These curves were generated programmatically using Python 3.10 with the matplotlib library.

### 3.11. Quality Regression Module

We adopt a multi-layer Graph Convolutional Network (GCN) as the regression model to predict perceptual quality scores. The GCN effectively captures hierarchical spatial and structural relationships among the perceptual clusters identified in earlier stages. The model operates on the graph structure defined by the predicted adjacency matrix $Z_{\mathrm{pred}}$ from the GATF module, using the computed node degrees as initial features.

Our implementation consists of three sequential graph convolutional blocks, each designed to extract and refine features at increasing levels of abstraction. Our GCN solution uses the normalized adjacency matrix to spread and mix information across clusters, in contrast to attention-based methods. This design enables the model to capture both geometric and perceptual, while batch normalization and non-linearity refine feature representations at each stage. After three successive GCN blocks, a global max pooling operation condenses cluster-level embeddings into a compact global descriptor, which is passed through a final dense layer to regress the perceptual quality score.

The key advantages of our deep GCN-based approach include its hierarchical feature learning capability, which captures both local distortions and global structural integrity through successive layers of feature transformation and aggregation. The architecture demonstrates robust handling of the irregular graph structures inherent in point cloud data. Following the final convolutional block, a global max pooling layer generates a fixed-size graph-level representation by selecting the most relevant features across all nodes. This representation is subsequently transformed into a final quality score through a linear regression layer. Experimental results demonstrate that this architecture achieves strong correlation with human subjective scores by effectively distilling the structural information encoded in the graph into a holistic quality estimate.

## 4. Experiments

This section describes the experimental setup, including the databases, evaluation metrics, and implementation details. We further provide comparative performance analyses and ablation studies to validate the effectiveness of the proposed approach. The section concludes with a discussion of the results and final observations.

### 4.1. Validation Databases

The efficacy of the proposed approach is validated through experiments on three publicly accessible databases: SJTU-PCQA [81], WPC [82], and ICIP2020 [83].

The SJTU-PCQA database comprises 10 reference point clouds, from which 420 distorted versions are derived using seven distortion types across six levels. Among these, 9 reference point clouds and 378 associated distorted versions are publicly available. The distortions comprise color noise (CN), Octree-based compression (OT), downscaling (DS), Gaussian geometry noise (GGN), downscaling combined with Gaussian geometry noise (D + G), color noise combined with Gaussian geometry noise (C + G), and downscaling combined with color noise (D + C). Subjective evaluation is provided as MOS values in the range [1, 10].

The WPC database consists of 20 original references and 740 distorted versions produced via five distortion schemes: Gaussian noise (GN), G-PCC (Octree), downsampling (DS), G-PCC (Trisoup), and V-PCC. The subjective scores are MOS values ranging from 0 to 100.

**Figure 5:**
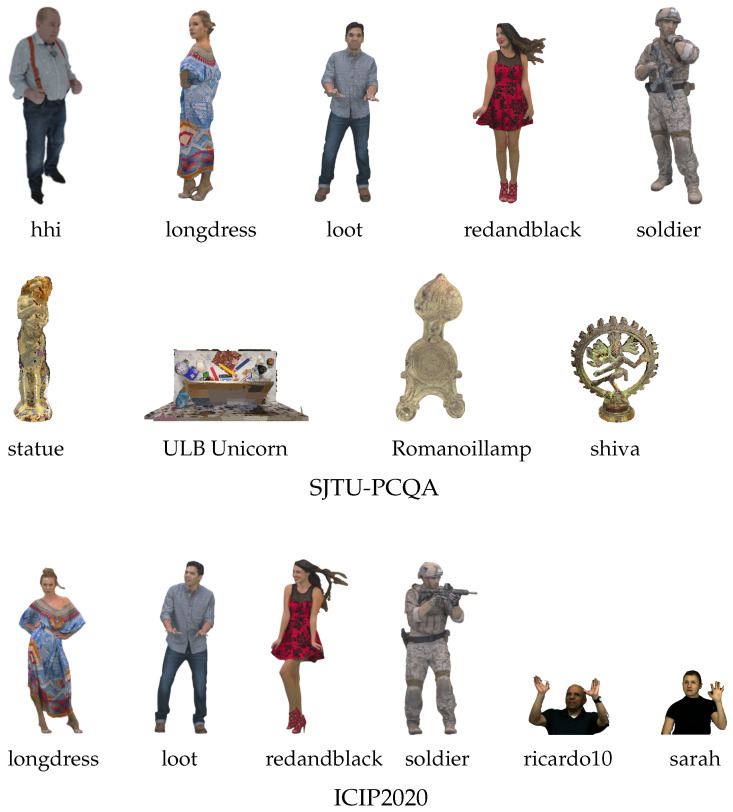
Reference point cloud samples from the SJTU-PCQA [81] and ICIP2020 [83] databases.

**Figure 6:**
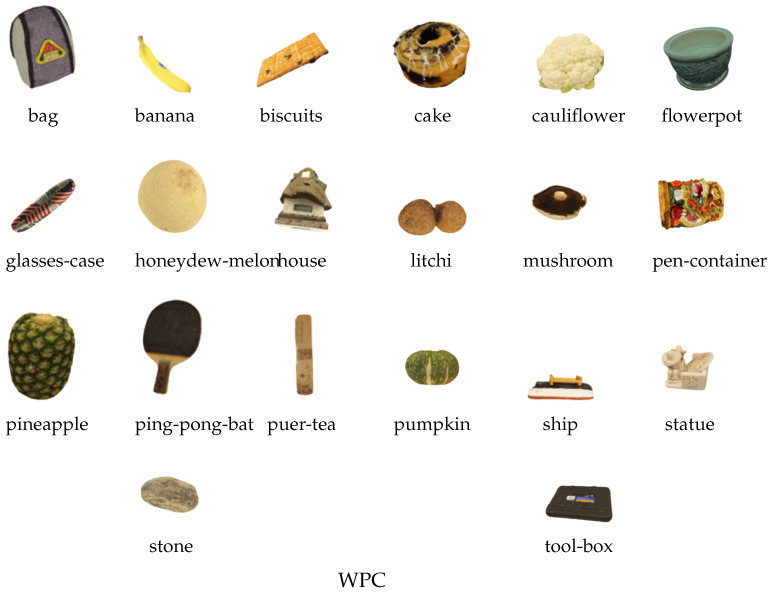
Reference point cloud samples from WPC [82] dataset.

The ICIP2020 database contains six reference point clouds with both geometry and texture, along with 90 distorted versions generated using VPCC, G-PCC Trisoup, and G-PCC Octree, each applied at five different quality levels. Representative reference samples from the three databases are shown in Figure 5 and Figure 6.

### 4.2. Evaluation Criteria

The performance of point cloud quality assessment metrics is commonly evaluated using four standard criteria: the Spearman Rank Correlation Coefficient (SRCC), Pearson Linear Correlation Coefficient (PLCC), Kendall Rank Correlation Coefficient (KRCC), and Root Mean Squared Error (RMSE). A high-quality model is characterized by PLCC, SRCC, and KRCC values close to 1, and an RMSE value near 0. To align predicted scores with subjective assessments, a five-parameter logistic mapping function is applied [84]:

(32) $${\tilde{s}}_{i}=\gamma_{1}\left(0.5-\frac{1}{1+\exp\left(\gamma_{2}\left(s_{i}-\gamma_{3}\right)\right)}\right)+\gamma_{4}s_{i}+\gamma_{5},$$

where $s_{i}$ denotes the raw predicted score for the *i*-th sample, ${\tilde{s}}_{i}$ is the mapped score, and $\gamma_{j}$ (j=1,2,…,5) are the fitted logistic parameters.

### 4.3. Comparative Performance Analysis

In this study, we benchmark our proposed method against 28 state-of-the-art point cloud quality assessment (PCQA) approaches, which we categorize by their methodology. The compared methods include point-based metrics that operate directly on 3D geometry and attributes. This category includes PSNR variants (e.g., PSNRmse,p2po [51], PSNRmse,p2pl [3]) which use point-to-point or point-to-plane distances, along with Angular Similarity measures (ASmean, ASrms, ASmse) [53], PSNR*Y* [52] in the luminance domain, and feature-based methods like PCQM [4], PointSSIM [57], and GraphSIM [60]. We also evaluate projection-based metrics that render 3D data into 2D views for analysis. This group includes classical image quality metrics such as SSIM [2], its multi-scale version MS-SSIM [37], the information-weighted IW-SSIM [38], and VIFP [39]. It also covers reduced-reference methods, including PCMRR [7] and RR-CAP [42]. Finally, we compare against a comprehensive set of no-reference (NR) learning-based models. These NR models span point-based approaches such as 3D-NSS [12], GPA-Net [66], ResSCNN [11], and PAME [65]. The NR category also includes projection-based models like PQA-Net [8], IT-PCQA [20], CoPA [48], OP-HPVS [49], DisPA [50], and GMS-3DQA [46], as well as the multi-modal method MM-PCQA [47]. Performance comparisons on the SJTU-PCQA, WPC, and ICIP2020 datasets are presented in Table 2, Table 3 and Table 4. Our method demonstrates state-of-the-art performance, achieving superior or highly competitive results across all three datasets, WPC, SJTU-PCQA, and ICIP2020, when compared to a comprehensive suite of full-reference (FR), reduced-reference (RR), and no-reference (NR) metrics.

On the SJTU-PCQA dataset, the method establishes a new benchmark for NR metrics, attaining the highest scores in PLCC (0.9480) and SRCC (0.9311), which are the primary indicators of prediction accuracy and monotonicity, respectively. It also secures first place in KRCC (0.7849) and a strong second place in RMSE (0.7843), being outperformed only marginally by MM-PCQA (0.7716). This performance is particularly significant as it surpasses all projection-based NR methods, including the recent and highly competitive MM-PCQA and GMS-3DQA, which were the previous state of the art.

On the WPC dataset, the results are exceptionally compelling. The proposed method achieves the highest rank in SRCC (0.8651) and KRCC (0.6827), indicating an unparalleled ability to correctly rank point clouds according to their perceptual quality. It places third in PLCC (0.8230) and RMSE (12.6914). The highly competitive nature of the top results on this dataset with MM-PCQA, GMS-3DQA, and the proposed method each leading in different metrics highlights the challenge it presents and confirms that the proposed method is among the elite performers.

The most dominant performance is observed on the ICIP2020 dataset. The proposed method delivers a near-perfect correlation with human opinion scores, achieving outstanding results: PLCC (0.9829), SRCC (0.9878), KRCC (0.9555), and RMSE (0.1950). It significantly outperforms all other listed methods, including the top FR metrics like PCQM (PLCC: 0.942, SRCC: 0.977) and ${\mathrm{PSNR}}_{mse,p2pl}$ (PLCC: 0.913). This remarkable achievement demonstrates that the model not only predicts subjective quality but does so with an accuracy that begins to approach the theoretical limit, effectively closing the gap between objective and subjective evaluation on this dataset.

A statistical comparison with leading NR-PCQA methods confirms the significant superiority of our approach. It outperforms the 2nd-ranked MM-PCQA on SJTU with absolute gains of +0.0254 PLCC and +0.0208 SRCC, while achieving a decisive +0.0237 SRCC advantage on the challenging WPC dataset. The proposed model also shows substantial improvements over other top methods: +0.0303 PLCC/+0.0210 SRCC over GMS-3DQA, +0.0290 PLCC/+0.0231 SRCC over DisPA, and most notably, +0.0430 PLCC/+0.0421 SRCC over the PC-based PAME. These consistent margins across multiple datasets and metrics confirm that our graph-based approach establishes a new state-of-the-art in native 3D quality assessment, effectively bridging the performance gap between point cloud-based and projection-based paradigms.

Since point cloud quality databases include a variety of content and distortions, it is important to assess PCQA methods under different content and distortion scenarios. The corresponding experimental outcomes for the SJTU-PCQA and WPC datasets are provided in Table 5 and Table 6 for SJTU-PCQA, and Table 7, Table 8, and Table 9 for WPC, respectively.

For the SJTU-PCQA dataset (Table 5), a content-based analysis shows that the proposed method exhibits remarkable consistency. It achieves first-place SRCC rankings in five of the nine content categories (Hhi, Longdress, Loot, Redandblack, ULB Unicorn) and secures a position within the top three in two others (Soldier, Statue). This indicates a low dependency on specific semantic features, textures, or geometric complexities inherent to different point clouds. The method’s only notable deviation occurs on the “Romanoillamp” and “Shiva” sequences, where it places outside the top three. This performance dip, shared by many other methods, suggests that these specific sequences may contain unique perceptual attributes or complex distortion interactions that are not yet fully captured by existing models, presenting a valuable direction for future research.

Considering the distortion-specific analysis (Table 6), our approach achieves first place in SRCC across all seven distortion categories with a significant margin. This performance is especially impressive for difficult distortion types like downsampling (DS: 0.9330 vs. 0.8654), color noise (CN: 0.8857 vs. 0.7779), and Octree-based compression (OT: 0.8285 vs. 0.7108), where it significantly surpasses the second-place method. From geometric artifacts (DS, D + G) to photometric impairments (CN, GGN) and complicated mixed distortions (D + C, C + G), this exhibits an unmatched capacity to generalize across fundamentally distinct distortion features. Its superior performance on both pure and mixed distortions indicates that its graph-based method successfully separates and assesses the intricate interaction between geometric and colorimetric information, a task that other approaches especially projection-based ones frequently find difficult.

On the WPC dataset (Table 7 and Table 8), the content-based evaluation, encompassing twenty distinct objects, shows that the proposed method achieves exceptional consistency. It secures a position within the top three ranking methods in sixteen of the twenty categories. More significantly, it attains the highest SRCC value in seven categories (e.g., Banana, Cauliflower, House, ToolBox) and ranks second in an additional five. Instead, the method successfully learns a generalized representation of perceptual quality that transfers effectively across a wide spectrum of content. Isolated underperformance on a few objects (e.g., HoneydewMelon, PuerTea) is a challenge shared across most metrics, suggesting these sequences may contain particularly complex or ambiguous perceptual attributes.

Examining various types of distortion (Table 9), our approach achieves first or second place in four out of five distortion categories, demonstrating a decisive advantage in handling diverse impairment types. Notably, it delivers state-of-the-art performance on the challenging G-PCC (Octree) compression distortion (1st place, 0.9073) and downsampling (2nd place, 0.8738). This is a critical advancement, as these geometric distortions are particularly prevalent and destructive. The method’s ability to excel on both pure noise additions (Gaussian Noise, 3rd place) and complex compression artifacts (V-PCC, 2nd place; G-PCC, 1st) underscores its capacity to evaluate the interplay between geometric and colorimetric information effectively.

Overall, the comprehensive evaluation across the SJTU and WPC databases demonstrates that our proposed GATF method systematically and significantly outperforms existing state-of-the-art approaches. By achieving superior and highly consistent SRCC performance across a vast array of diverse content and distortion categories, our method proves its exceptional robustness and generalizability. These results underscore the effectiveness of the Graph Attention Transformer Fusion architecture in capturing perceptually relevant features and structural relationships within point clouds, establishing a new benchmark for a precise and reliable evaluation of the no-reference point cloud quality.

Although our method achieves state-of-the-art performance, its limitations are revealed through detailed analysis. Optimal outcomes occur for mid-density point clouds with 500 K to 1 M points, such as the Statue model with an SRCC of 0.9000, with slight degradation on ultra-dense models like ULB Unicorn with 2M points, SRCC = 0.9167, indicating potential computational scalability issues. Performance is also influenced by semantic content, excelling on textured objects like Longdress (SRCC = 0.9662) but less effective on homogeneous surfaces like Romanoillamp (SRCC = 0.7665), suggesting a dependency on strong visual features. Major limitations include sensitivity to extreme density variations, variable robustness across content types, and possible dependence on textural complexity. This evaluation precisely maps the method’s boundaries, highlighting the need for future research to enhance generalization for sparse, ultra-dense, and texture-less point clouds, thereby advancing the broader applicability of deep learning-based PCQA methods.

### 4.4. Ablation Study

To assess the individual contribution of each perceptual feature and the efficacy of the Graph Attention Network Transformer Fusion (GATF) module, a comprehensive ablation study was performed. The performance of each feature was evaluated both in isolation and in various combinations to quantify its impact on prediction accuracy. Crucially, the role of the GATF mechanism was examined by comparing its attentive fusion against a simple averaging strategy of the unimodal scores. The results, detailed in Table 10, demonstrate the significance of each feature curvature (Curv), saliency (Sal), and color (LAB) and conclusively highlight the superior performance of the learned, attentive fusion provided by GATF compared to a simple merge approach that calculates the average of scores.

Analyzing the unimodal features reveals a clear performance hierarchy. Across all three databases, the saliency (SAL) feature consistently emerges as the single most powerful predictor, attaining the greatest SRCC and PLCC scores individually (SJTU-PCQA: SRCC = 0.8491, PLCC = 0.8550; WPC: SRCC = 0.7121, PLCC = 0.7395; ICIP2020: SRCC = 0.9215, PLCC = 0.9263). This suggests that human perceptual judgment of point cloud quality is most strongly influenced by regions of visual attention. The curvature (CURV) feature consistently ranks second, outperforming the LAB color feature on the SJTU-PCQA and ICIP2020 datasets (for example, ICIP2020: SRCC = 0.9151, PLCC = 0.9006), which underscores the fundamental importance of geometric integrity over color fidelity in quality perception. The LAB color space, while the weakest standalone performer, still provides a substantial baseline, indicating that color distortions are perceptually relevant (for instance, WPC: PLCC = 0.7056, SRCC = 0.6859), but less critical than geometric ones.

The evaluation of feature combinations without GATF exposes the severe limitations of a simple fusion strategy. In every case, simply averaging the scores results in suboptimal performance that fails to capitalize on the potential synergies between features. For instance, combining LAB and CURV without GATF on the WPC dataset yields a PLCC of 0.6921 and SRCC of 0.6658, which is not only worse than the SAL feature alone but also worse than the LAB feature individually. This indicates that a linear fusion can dilute strong features and introduce noise, failing to model the complex interdependencies between perceptual attributes.

The introduction of the GATF mechanism is the pivotal element that unlocks transformative performance gains. In every single combination, the GATF-based model delivers a substantial improvement over its non-GATF counterpart. The most profound result is achieved by the CURV + SAL with GATF configuration, which attains state-of-the-art performance across the board: on SJTU-PCQA (SRCC = 0.9311, PLCC = 0.9480), WPC (SRCC = 0.8651, PLCC = 0.8030), and near-perfect correlation on ICIP2020 (SRCC = 0.9878, PLCC = 0.9829). This demonstrates that the most critical information resides in the geometric and saliency domains, and the GATF is exceptionally capable of fusing them into a powerfully predictive representation. The full LAB + CURV + SAL with GATF model also performs exceptionally, securing second place on SJTU-PCQA (PLCC = 0.8979, SRCC = 0.9005), ICIP2020 (SRCC = 0.9757, PLCC = 0.9754), and WPC (SRCC = 0.8238, PLCC = 0.7981). This confirms that while color is less critical, the GATF can still effectively integrate it as a complementary signal to enhance robustness, though its addition in this case slightly dilutes the optimal fusion achieved by CURV + SAL alone.

The findings highlight the need to combine several perceptual characteristics in order to attain reliable and broadly applicable performance. Additionally, they unequivocally show how crucial the Graph Attention Network Transformer Fusion (GATF) mechanism is to accurately simulating intricate distortion properties and guaranteeing reliable quality forecasts across various datasets. Together, these results demonstrate the importance of GATF and multi-feature integration in improving the precision and dependability of point cloud quality evaluation.

### 4.5. Cross-Database Validation

The experimental results for cross-database evaluation reveal the generalization ability of the proposed model across different datasets. Specifically, the setting WPC→SJTU corresponds to training the model on the WPC database and subsequently testing it on the SJTU-PCQA database, while the reverse setting SJTU→WPC indicates training on the SJTU-PCQA database and testing on the WPC database. The experimental results are presented in Table 11.

The proposed method demonstrates superior cross-database robustness, achieving the highest performance in both directions: WPC→SJTU (SRCC = 0.7615, PLCC = 0.7902) and SJTU→WPC (SRCC = 0.5144, PLCC = 0.5099). This indicates that the features learned by the proposed model are more invariant to database-specific characteristics than those of other methods. The performance of other models varies considerably. Methods like MM-PCQA and GMS-3DQA show strong generalization in the WPC→SJTU direction (PLCC: 0.7403 and 0.7611, respectively) but suffer from a catastrophic drop in the reverse SJTU→WPC scenario (PLCC: ∼0.30), suggesting their learned representations may be overly tailored to the features of their primary training set. Earlier approaches like 3D-NSS and ResSCNN show poor generalization in both directions, with SRCC values often falling below 0.25, confirming that simpler or less expressive models fail to learn transferable perceptual representations. Overall, these findings highlight that even though intra-database evaluation is inherently superior to cross-database performance, the model is able to maintain strong correlations with subjective scores through the use of GATF and the integration of perceptual features, guaranteeing accurate predictions in a variety of database scenarios.

### 4.6. Computational Efficiency

The practical deployment of a point cloud quality assessment (PCQA) method hinges not only on its prediction accuracy but also on its computational efficiency. This is particularly critical for our approach, which performs analysis directly on the 3D point cloud data. To evaluate this, we conducted a comparative analysis of the average inference time per point cloud between our proposed method and several leading model-based benchmarks, including 3D-NSS, GraphSIM, PCMRR, PCQM, and PointSSIM.

**Figure 7:**
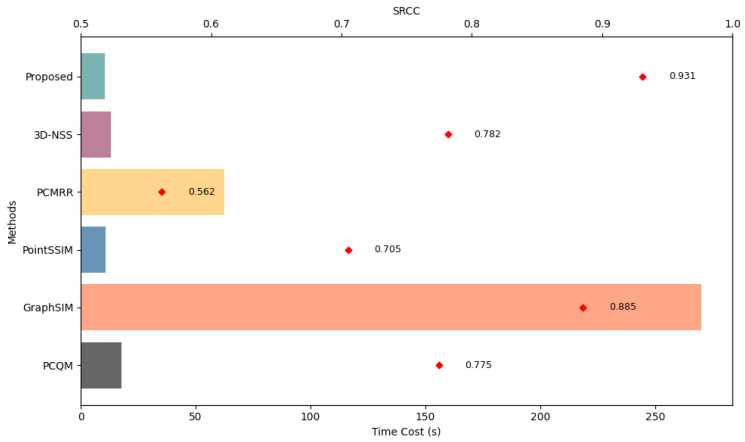
Comparison of average processing time and SRCC performance on the SJTU-PCQA database.

The results in Figure 7 demonstrate a key strength of our framework: it achieves superior performance without a computational penalty. Our method attains the highest SRCC (0.931) on the SJTU-PCQA dataset while also recording the lowest inference time. Quantitatively, it is 25.8 times faster than GraphSIM (270.14 s) and marginally faster than PointSSIM (10.63 s), proving that our architecture effectively circumvents the typical accuracy–speed trade-off. The efficiency stems from our streamlined graph processing pipeline, which avoids the computational overhead of complex feature aggregation found in other methods. This combination of state-of-the-art accuracy and superior computational efficiency makes our model highly suitable for real-world applications requiring fast and reliable quality assessment, such as in compression pipelines or real-time streaming systems.

The suggested approach was put into practice on a Windows computer (Dell, France) that has an Intel Core i7-11800H @ 2.30 GHz processor, 32 GB of RAM, and an NVIDIA GeForce RTX 3060 laptop GPU (6 GB VRAM).

## 5. Discussion

This section analyzes the results and explains the behavior of the proposed method from three perspectives: accuracy, robustness, computational efficiency and limitations.

### 5.1. Overall Performance

The proposed method demonstrates exceptional performance across all three datasets. In the case of SJTU-PCQA, it achieves the highest values for PLCC (0.9480) and SRCC (0.9311). For WPC, it records the top SRCC (0.8651) and KRCC (0.6827) scores. On ICIP2020, it showcases an almost perfect correlation with human assessments, with PLCC (0.9829) and SRCC (0.9878). These findings validate that the model effectively captures both monotonicity and accuracy, regardless of the content and types of distortions present. Additionally, it outperforms robust projection-based approaches like MM-PCQA and GMS-3DQA, highlighting the advantages of its native 3D processing capabilities.

### 5.2. Robustness to Content and Distortion

The content-based analysis shows consistent top-three rankings across most point clouds. The method performs well on both simple and complex geometry, indicating low dependency on specific content. For distortion-based analysis, it ranks first in all distortion types, including geometric (DS, D + G), photometric (CN, GGN), and mixed distortions (D + C, C + G). This demonstrates that the graph-based representation and feature fusion handle both local and global degradation patterns effectively.

### 5.3. Computational Efficiency and Limitations

Computational efficiency is central to the deployment of PCQA metrics. While the method involves multi-stage processing, including perceptual clustering, graph construction, and Graph Attention Transformer Fusion (GATF), the proposed method remains suitable for real-time use. Clustering and graph generation are executed once per point cloud and leverage GPU acceleration. The GATF module, though complex, benefits from parallel GPU processing during inference. Using clustered regions instead of individual points reduces complexity while preserving perceptual accuracy. On mid-range hardware, the approach achieves near real-time performance, making it promising for interactive systems requiring fast and accurate quality assessment.

However, the analysis also reveals specific limitations. Performance, while consistently high, shows some variance depending on the semantic content and density of the point cloud. For instance, the model excels on textured, complex objects like Longdress but is less effective on homogeneous surfaces like Romanoillamp. This suggests a degree of dependency on strong visual and geometric features, indicating that the model’s perceptual clustering may be less effective for content with low textural or geometric variation. Furthermore, while robust across a range of densities, the slight performance degradation on ultra-dense models (e.g., ULB Unicorn) points to potential computational scalability challenges that must be addressed for applications involving very large-scale point clouds.

## 6. Conclusions

This work introduced a novel graph-based learning framework for no-reference point cloud quality assessment that effectively bridges the gap between objective prediction and human perception. By jointly modeling perceptual characteristics like curvature, saliency, and color within a unified graph representation, the proposed approach transforms irregular 3D data into a structured form amenable to deep learning. The incorporation of the Graph Attention Network Transformer Fusion (GATF) module represents a key innovation, as it enables dynamic refinement of feature importance and adaptive fusion across modalities. Coupled with a Graph Convolutional Network for final regression, this architecture provides a principled mechanism to capture both local geometric degradations and global perceptual cues.

Extensive experiments on three widely used benchmarks, WPC, SJTU-PCQA, and ICIP2020, demonstrate the effectiveness of the proposed method. The proposed model demonstrates consistently superior correlation with human subjective ratings, achieving higher PLCC and SRCC values than all state-of-the-art metrics across diverse distortion types and point cloud densities. These results confirm our framework’s capacity to accurately model both fine-grained distortions and high-level perceptual characteristics, highlighting its strong robustness and generalization ability across varying content and impairment conditions.

Several strategic research trajectories emerge to extend this work. Computational efficiency could be enhanced through lightweight super-voxel clustering and quantized network implementations. The framework’s distortion robustness should be expanded to include sensor-induced noise typical of real 3D acquisition systems and new compression schemes derived from emerging MPEG standards. Temporal modeling capabilities could be integrated via 4D spatiotemporal graphs to handle dynamic point cloud sequences. Pre-training using large-scale unlabeled 3D data with contrastive or masked modeling objectives would improve cross-dataset generalization. These advancements will preserve the fundamental perceptual alignment of the framework through its modular architecture, while greatly improving practical deployment in applications such as autonomous systems and volumetric video streaming.

Overall, the proposed framework provides a unified, perceptually aligned, and computationally feasible solution for point cloud quality assessment. By combining strong accuracy, robustness to diverse distortions, and practical efficiency, it advances the development of reliable NR-PCQA models and establishes a solid foundation for future research and real-world deployment.

## Figures and Tables

**Table 1:** Summary of perceptual features used in the proposed method.

Feature	Description	Mathematical Definition
**Color**	Perceptual color in CIELAB space, providing uniform color difference perception.	$C^{\mathrm{Lab}}\left(p_{i}\right)=\left(L_{i}^{*},a_{i}^{*},b_{i}^{*}\right)$ where $\left[\begin{array}{c} X_{i}\\ Y_{i}\\ Z_{i} \end{array}\right]=M\cdot \left[\begin{array}{c} R_{i}\\ G_{i}\\ B_{i} \end{array}\right]$ *M* is the transformation matrix. $L_{i}^{*}=116\cdot f\left(Y_{i}/Y_{n}\right)-16$ $a_{i}^{*}=500\cdot \left[f\left(X_{i}/X_{n}\right)-f\left(Y_{i}/Y_{n}\right)\right]$ $b_{i}^{*}=200\cdot \left[f\left(Y_{i}/Y_{n}\right)-f\left(Z_{i}/Z_{n}\right)\right]$ $f\left(t\right)=\left\{\begin{array}{cc} t^{1/3} & t>\delta^{3}\\ \frac{t}{3\delta^{2}}+\frac{16}{116} & \mathrm{otherwise} \end{array}\right.$ with $\delta =\frac{6}{29}$ $\left(X_{n},Y_{n},Z_{n}\right)$: reference white point
**Curvature**	Local surface deviation from flatness, capturing geometric details.	$Curv\left(p_{i}\right)=\frac{\mu_{3}}{\mu_{1}+\mu_{2}+\mu_{3}}$ where $\Sigma_{i}$ is the covariance matrix: $\Sigma_{i}=\frac{1}{\left|N(p_{i},r)\right|}\sum_{q_{k}\in N\left(p_{i},r\right)}\left(q_{k}-{\bar{q}}_{i}\right){\left(q_{k}-{\bar{q}}_{i}\right)}^{⊤}$ ${\bar{q}}_{i}=\frac{1}{\left|N(p_{i},r)\right|}\sum_{q_{k}\in N\left(p_{i},r\right)}q_{k}$ $\mu_{1}\geq \mu_{2}\geq \mu_{3}$: eigenvalues of $\Sigma_{i}$ $N(p_{i},r)$: local neighborhood with radius *r*
**Saliency**	Visual prominence indicating attention-attracting regions.	$Sal\left(p_{i}\right)=\left|n\left(p_{i}\right)\cdot \left(G_{\sigma_{1}}\left(p_{i}\right)-G_{\sigma_{2}}\left(p_{i}\right)\right)\right|$ where $n(p_{i})$ is surface normal, $G_{\sigma }$ are Gaussian-smoothed representations at scales $\sigma_{1}<\sigma_{2}$

**Table 2:** Comparison of performance on the SJTU-PCQA dataset. The arrows (↑ and ↓) indicate the desired direction of performance, where ↑ denotes higher values are better and ↓ denotes lower values are better. The top three scores for each criterion are highlighted in red, blue, and green for ranks one through three, respectively.

Ref	Type	Metric	SJTU-PCQA			
			PLCC ↑	SRCC ↑	KRCC ↑	RMSE ↓
FR	PC-Based	${\mathrm{PSNR}}_{mse,p2po}$ [51]	0.7622	0.6002	0.4917	1.4382
		${\mathrm{PSNR}}_{mse,p2pl}$ [3]	0.7381	0.5505	0.4375	1.5357
		${\mathrm{PSNR}}_{hf,p2po}$ [51]	0.7737	0.6744	0.5217	1.4481
		${\mathrm{PSNR}}_{hf,p2pl}$ [3]	0.7286	0.6208	0.4701	1.6000
		${\mathrm{AS}}_{mean}$ [53]	0.5297	0.5317	0.3723	2.7129
		${\mathrm{AS}}_{rms}$ [53]	0.7156	0.5653	0.4144	1.6550
		${\mathrm{AS}}_{mse}$ [53]	0.5115	0.5472	0.3865	2.6431
		${\mathrm{PSNR}}_{Y}$ [52]	0.8124	0.7871	0.6116	1.3222
		PCQM [4]	0.8653	0.8544	0.6152	1.2978
		PointSSIM [57]	0.7422	0.7051	0.5321	1.5601
		GraphSIM [60]	0.9158	0.8853	0.7063	0.9462
	Projection-Based	SSIM [2]	0.8868	0.8667	0.6988	1.0454
		MS-SSIM [37]	0.8930	0.8738	0.7069	1.0091
		IW-SSIM [38]	0.8932	0.8638	0.6934	1.0268
		VIFP [39]	0.8977	0.8624	0.6934	1.0173
RR	PC-Based	PCMRR [7]	0.6699	0.5622	0.4091	1.7589
	Projection-Based	RR-CAP [42]	0.7691	0.7577	0.5508	1.5512
NR	PC-Based	3D-NSS [12]	0.7813	0.7819	0.6023	1.7740
		GPA-Net [66]	0.8860	0.8750	-	-
		ResSCNN [11]	0.8865	0.8328	0.6514	1.0728
		PAME [65]	0.9050	0.8890	-	1.0100
	Projection-Based	PQA-Net [8]	0.7998	0.7593	0.5796	1.3773
		IT-PCQA [20]	0.8605	0.8286	0.6453	1.1686
		CoPA [48]	0.9130	0.8970	-	0.9200
		OP-HPVS [49]	0.9155	0.9041	-	0.9263
		DisPA [50]	0.9190	0.9080	-	0.8900
		GMS-3DQA [46]	0.9177	0.9101	0.7735	0.7872
		MM-PCQA [47]	0.9226	0.9103	0.7838	0.7716
	PC-Based	Proposed	0.9480	0.9311	0.7849	0.7843

**Table 3:** Comparison of performance on the WPC dataset. The arrows (↑ and ↓) indicate the desired direction of performance, where ↑ denotes higher values are better and ↓ denotes lower values are better. The top three scores for each criterion are highlighted in red, blue, and green for ranks one through three, respectively.

Ref	Type	Metric	WPC			
			PLCC ↑	SRCC ↑	KRCC ↑	RMSE ↓
FR	PC-Based	${\mathrm{PSNR}}_{mse,p2po}$ [51]	0.2673	0.1607	0.1147	20.6947
		${\mathrm{PSNR}}_{mse,p2pl}$ [3]	0.2879	0.1182	0.0851	21.1898
		${\mathrm{PSNR}}_{hf,p2po}$ [51]	0.3555	0.0557	0.0384	20.8197
		${\mathrm{PSNR}}_{hf,p2pl}$ [3]	0.3263	0.0989	0.0681	21.1100
		${\mathrm{AS}}_{mean}$ [53]	0.3397	0.2484	0.1801	21.5013
		${\mathrm{AS}}_{rms}$ [53]	0.3347	0.2479	0.1802	21.5325
		${\mathrm{AS}}_{mse}$ [53]	0.3397	0.2484	0.1801	21.5013
		${\mathrm{PSNR}}_{Y}$ [52]	0.6166	0.5823	0.4164	17.9001
		PCQM [4]	0.6162	0.5504	0.4409	17.9027
		PointSSIM [57]	0.5225	0.4639	0.3394	19.3863
		GraphSIM [60]	0.6833	0.6217	0.4562	16.5107
	Projection-Based	SSIM [2]	0.6690	0.6483	0.4685	16.8841
		MS-SSIM [37]	0.7349	0.7179	0.5385	15.3341
		IW-SSIM [38]	0.7688	0.7608	0.5707	14.5453
		VIFP [39]	0.7508	0.7426	0.5575	15.0328
RR	PC-Based	PCMRR [7]	0.3926	0.3605	0.2543	20.9203
	Projection-Based	RR-CAP [42]	0.7307	0.7162	0.5260	15.6485
NR	PC-Based	3D-NSS [12]	0.6284	0.6309	0.4573	18.1706
		GPA-Net [66]	0.7690	0.7580	-	-
		ResSCNN [11]	0.4531	0.4362	0.2987	20.2591
		PAME [65]	0.7810	0.7630	-	14.5000
	Projection-Based	PQA-Net [8]	0.6671	0.6368	0.4684	16.6758
		IT-PCQA [20]	0.4870	0.4329	0.3006	19.8960
		CoPA [48]	0.7850	0.7790	-	14.4000
		OP-HPVS [49]	0.8137	0.8329	-	12.9895
		DisPA [50]	0.7900	0.7880	-	13.8000
		GMS-3DQA [46]	0.8338	0.8308	0.6457	12.2292
		MM-PCQA [47]	0.8556	0.8414	0.6513	12.3506
	PC-Based	Proposed	0.8230	0.8651	0.6827	12.6914

**Table 4:** Comparison of performance on the ICIP2020 dataset. The arrows (↑ and ↓) indicate the desired direction of performance, where ↑ denotes higher values are better and ↓ denotes lower values are better. The top three scores for each criterion are highlighted in red, blue, and green for ranks one through three, respectively.

Ref	Type	Metric	ICIP2020			
			PLCC ↑	SRCC ↑	KRCC ↑	RMSE ↓
FR	PC-Based	${\mathrm{PSNR}}_{mse,p2po}$ [51]	0.8826	0.8914	-	0.5229
		${\mathrm{PSNR}}_{mse,p2pl}$ [3]	0.913	0.915	-	0.463
		${\mathrm{PSNR}}_{hf,p2po}$ [51]	0.601	0.542	-	0.908
		${\mathrm{PSNR}}_{hf,p2pl}$ [3]	0.649	0.602	-	0.865
		${\mathrm{AS}}_{mean}$ [53]	-	-	-	-
		${\mathrm{AS}}_{rms}$ [53]	-	-	-	-
		${\mathrm{AS}}_{mse}$ [53]	-	-	-	-
		${\mathrm{PSNR}}_{Y}$ [52]	0.7539	0.7388	-	0.7307
		PCQM [4]	0.942	0.977	-	0.518
		PointSSIM [57]	0.904	0.865	-	0.486
		GraphSIM [60]	0.890	0.872	-	0.518
	Projection-Based	SSIM [2]	-	-	-	-
		MS-SSIM [37]	-	-	-	-
		IW-SSIM [38]	0.8919	0.8608	-	0.5029
		VIFP [39]	-	-	-	-
RR	PC-Based	PCMRR [7]	0.7586	0.8463	-	1.1122
	Projection-Based	RR-CAP [42]	-	-	-	-
NR	PC-Based	3D-NSS [12]	-	-	-	-
		GPA-Net [66]	-	-	-	-
		ResSCNN [11]	-	-	-	-
		PAME [65]	-	-	-	-
	Projection-Based	PQA-Net [8]	-	-	-	-
		IT-PCQA [20]	-	-	-	-
		CoPA [48]	-	-	-	-
		OP-HPVS [49]	-	-	-	-
		DisPA [50]	-	-	-	-
		GMS-3DQA [46]	-	-	-	-
		MM-PCQA [47]	0.7240	0.5933	-	0.7672
	PC-Based	Proposed	0.9829	0.9878	0.9555	0.1950

**Table 5:** SRCC performance of various PCQA metrics across different point cloud contents in the SJTU-PCQA database. The top three results for each content type are highlighted in red (1st), blue (2nd), and green (3rd).

Metric	Hhi	Longdress	Loot	Redand	Romanoi	Shiva	Soldier	Statue	ULB
${\mathrm{PSNR}}_{mse,p2po}$ [51]	0.6526	0.6640	0.6738	0.6196	0.4247	0.4129	0.6781	0.5678	0.7085
${\mathrm{PSNR}}_{mse,p2pl}$ [3]	0.5150	0.6437	0.6405	0.5943	0.3617	0.4074	0.6478	0.5362	0.6082
${\mathrm{PSNR}}_{hf,p2po}$ [51]	0.7443	0.7885	0.7447	0.7421	0.7457	0.1168	0.7493	0.5883	0.8500
${\mathrm{PSNR}}_{hf,p2pl}$ [3]	0.6785	0.7096	0.6391	0.6819	0.6032	0.2689	0.6329	0.5652	0.8081
${\mathrm{AS}}_{rms}$ [53]	0.5012	0.5704	0.4817	0.5799	0.6022	0.7057	0.5404	0.6291	0.4773
${\mathrm{PSNR}}_{Y}$ [52]	0.8242	0.9326	0.7875	0.7478	0.4278	0.8375	0.8336	0.8241	0.8687
PCQM [4]	0.7524	0.8896	0.8426	0.8024	0.5145	0.8060	0.8684	0.7483	0.7496
PointSSIM [57]	0.7010	0.8608	0.7299	0.6670	0.5150	0.7896	0.7718	0.7391	0.5715
GraphSIM [60]	0.9028	0.9499	0.8868	0.8702	0.8525	0.8595	0.9118	0.8744	0.8597
SSIM [2]	0.8409	0.9245	0.8693	0.8603	0.7509	0.8968	0.8917	0.8578	0.9084
MS-SSIM [37]	0.8658	0.9191	0.8809	0.8718	0.7869	0.8914	0.8843	0.8663	0.8981
IW-SSIM [38]	0.8773	0.8710	0.8846	0.8911	0.7939	0.8744	0.8843	0.8428	0.8548
VIFP [39]	0.8462	0.8976	0.8619	0.8885	0.7882	0.8903	0.8744	0.8637	0.8514
PCMRR [7]	0.4785	0.6474	0.6770	0.6506	0.6044	0.4884	0.5809	0.4181	0.5148
3D-NSS [12]	0.7394	0.9005	0.8890	0.8647	0.6885	0.8198	0.8731	0.8520	0.4101
ResSCNN [11]	0.8240	0.8650	0.8780	0.8003	0.6193	0.8599	0.9123	0.9002	0.8364
PQA-Net [8]	0.8409	0.9245	0.8693	0.8603	0.7509	0.8968	0.8917	0.8578	0.9084
IT-PCQA [20]	0.7577	0.8243	0.8778	0.8557	0.7248	0.8243	0.8050	0.8757	0.9129
Proposed	0.9166	0.9662	0.9288	0.9334	0.7665	0.8500	0.9090	0.9000	0.9167

**Table 6:** SRCC results of PCQA metrics on the SJTU-PCQA database across various distortion types. The top three results are highlighted in red (1st), blue (2nd), and green (3rd).

Types	Metric	OT	CN	DS	D + C	D + G	GGN	C + G
FR	${\mathrm{PSNR}}_{mse,p2po}$ [51]	0.4407	NaN	0.4495	0.5735	0.6779	0.7008	0.7577
	${\mathrm{PSNR}}_{mse,p2pl}$ [3]	0.4407	NaN	0.4489	0.5979	0.7058	0.7144	0.7758
	${\mathrm{PSNR}}_{hf,p2po}$ [51]	0.3788	NaN	0.6847	0.7619	0.7423	0.7453	0.8205
	${\mathrm{PSNR}}_{hf,p2pl}$ [3]	0.3524	NaN	0.3286	0.7499	0.7196	0.7328	0.8025
	${\mathrm{AS}}_{rms}$ [53]	0.5210	NaN	0.3653	0.4025	0.8915	0.9376	0.9241
	${\mathrm{PSNR}}_{Y}$ [52]	0.3068	0.5588	0.4697	0.7397	0.5413	0.5727	0.6692
	PCQM [4]	0.6495	0.6070	0.6990	0.8014	0.7476	0.7143	0.7078
	PointSSIM [57]	0.7108	0.7660	0.8500	0.7449	0.9288	0.9027	0.7991
	GraphSIM [60]	0.7049	0.7779	0.8654	0.8846	0.8833	0.9064	0.9334
	SSIM [2]	0.2198	0.6283	0.3246	0.5062	0.6920	0.7436	0.7307
	MS-SSIM [37]	0.2712	0.6453	0.4718	0.6281	0.7589	0.7783	0.7948
	IW-SSIM [38]	0.3382	0.7531	0.4535	0.6661	0.8222	0.8324	0.8406
	VIFP [39]	0.3743	0.7429	0.4546	0.6932	0.7989	0.8436	0.8463
RR	PCMRR [7]	0.1800	0.7157	0.1489	0.6120	0.7439	0.7813	0.8329
NR	3D-NSS [12]	0.4068	0.1480	0.5051	0.5895	0.7442	0.8435	0.8645
	ResSCNN [11]	0.1683	0.2265	0.4292	0.5158	0.5263	0.4497	0.5523
	PQA-Net [8]	0.0883	0.5507	0.2958	0.4899	0.5033	0.3771	0.6137
	IT-PCQA [20]	0.0189	0.0655	0.0556	0.0468	0.0411	0.0798	0.1044
	Proposed	0.8285	0.8857	0.9330	0.9428	0.9610	0.9774	0.9541

**Table 7:** SRCC performance of various PCQA metrics across different point cloud contents in the WPC database (Part 1: Bag to Litchi). The top three results for each content type are highlighted in red (1st), blue (2nd), and green (3rd).

Metric	Bag	Ban	Bisc	Cake	Caul	Flow	Glass	Melon	House	Litch
${\mathrm{PSNR}}_{mse,p2po}$ [51]	0.6669	0.6471	0.5252	0.3074	0.3501	0.6509	0.5845	0.4890	0.5866	0.5109
${\mathrm{PSNR}}_{mse,p2pl}$ [3]	0.5751	0.5691	0.4160	0.1798	0.2058	0.5298	0.4390	0.3299	0.4483	0.4291
${\mathrm{PSNR}}_{hf,p2po}$ [51]	0.4363	0.1933	0.3085	0.1724	0.0918	0.4348	0.2020	0.2768	0.3429	0.3478
${\mathrm{PSNR}}_{hf,p2pl}$ [3]	0.4365	0.2033	0.3368	0.1796	0.1653	0.4515	0.3238	0.2300	0.3434	0.3204
${\mathrm{AS}}_{rms}$ [53]	0.4325	0.3147	0.3505	0.0609	0.1781	0.3629	0.4288	0.3228	0.4522	0.3554
${\mathrm{PSNR}}_{Y}$ [52]	0.8051	0.6211	0.7764	0.5180	0.5927	0.6385	0.7826	0.6740	0.7798	0.7027
PCQM [4]	0.5955	0.4649	0.6245	0.4566	0.4903	0.5875	0.5861	0.4500	0.5880	0.5965
PointSSIM [57]	0.4829	0.2202	0.5816	0.3177	0.4237	0.3784	0.5258	0.5609	0.5590	0.6422
GraphSIM [60]	0.7164	0.5045	0.7198	0.4251	0.5529	0.6609	0.6546	0.7248	0.7373	0.6958
SSIM [2]	0.7300	0.8011	0.9173	0.7390	0.8004	0.8303	0.7617	0.8549	0.7788	0.7748
MS-SSIM [37]	0.7584	0.7677	0.9500	0.7691	0.8608	0.9066	0.7577	0.8917	0.7793	0.8623
IW-SSIM [38]	0.7309	0.7790	0.7992	0.6534	0.8182	0.9047	0.7304	0.9180	0.7357	0.7496
VIFP [39]	0.7093	0.7771	0.7416	0.6477	0.7008	0.8954	0.7459	0.8279	0.7200	0.7018
PCMRR [7]	0.6069	0.5287	0.4310	0.3070	0.4187	0.0477	0.3883	0.5742	0.4905	0.4839
3D-NSS [12]	0.7731	0.6524	0.6645	0.4547	0.5517	0.6958	0.4790	0.7229	0.7646	0.8113
ResSCNN [11]	0.1603	0.2475	0.4765	0.4467	0.5095	0.4900	0.2003	0.4026	0.4780	0.1994
PQA-Net [8]	0.3504	0.6949	0.6147	0.5835	0.6238	0.2357	0.7674	0.7418	0.8668	0.7207
IT-PCQA [20]	0.6174	0.2485	0.3570	0.7300	0.0593	0.8127	0.7380	0.7352	0.4201	0.0868
Proposed	0.7619	0.8095	0.8571	0.7857	0.8809	0.9047	0.7857	0.7142	0.8832	0.8279

**Table 8:** SRCC performance of various PCQA metrics across different point cloud contents in the WPC database (Part 2: Mushroom to Tool). The top three results for each content type are highlighted in red (1st), blue (2nd), and green (3rd).

Metric	Mush	Pen	Pine	Bat	Tea	Pump	Ship	Stat	Stone	Tool
${\mathrm{PSNR}}_{mse,p2po}$ [51]	0.6396	0.7720	0.3777	0.5924	0.6069	0.4947	0.7464	0.8040	0.6219	0.3937
${\mathrm{PSNR}}_{mse,p2pl}$ [3]	0.5156	0.6688	0.2785	0.4984	0.4746	0.3423	0.6267	0.6707	0.5129	0.2969
${\mathrm{PSNR}}_{hf,p2po}$ [51]	0.3486	0.2159	0.1376	0.4958	0.1173	0.3092	0.3404	0.2450	0.3551	0.1972
${\mathrm{PSNR}}_{hf,p2pl}$ [3]	0.3105	0.3635	0.1831	0.4357	0.0384	0.3068	0.5158	0.4487	0.3424	0.1884
${\mathrm{AS}}_{rms}$ [53]	0.2911	0.5465	0.2155	0.4521	0.4734	0.3220	0.4943	0.4900	0.3649	0.2984
${\mathrm{PSNR}}_{Y}$ [52]	0.6550	0.7328	0.7217	0.5428	0.7639	0.6901	0.7786	0.7001	0.7115	0.8706
PCQM [4]	0.5725	0.6394	0.6427	0.5783	0.5685	0.5934	0.5434	0.5714	0.6475	0.6304
PointSSIM [57]	0.5443	0.5948	0.5386	0.6051	0.4139	0.5699	0.4488	0.5085	0.6126	0.4927
GraphSIM [60]	0.6802	0.8250	0.6401	0.7697	0.7999	0.6517	0.7558	0.7390	0.1920	0.7935
SSIM [2]	0.7821	0.8954	0.7307	0.8054	0.8917	0.9111	0.8973	0.8985	0.8426	0.7821
MS-SSIM [37]	0.8781	0.8758	0.7805	0.8812	0.8668	0.8156	0.8578	0.9372	0.8881	0.8255
IW-SSIM [38]	0.8160	0.8485	0.5856	0.7570	0.8359	0.9042	0.8340	0.9099	0.8587	0.8056
VIFP [39]	0.7897	0.8397	0.6441	0.7539	0.7866	0.8976	0.8013	0.8950	0.8196	0.7411
PCMRR [7]	0.2556	0.6830	0.4011	0.5092	0.4308	0.3241	0.4400	0.1811	0.3632	0.5239
3D-NSS [12]	0.8153	0.7809	0.6074	0.6935	0.4763	0.5768	0.6935	0.6368	0.6968	0.5806
ResSCNN [11]	0.0754	0.5676	0.5275	0.3518	0.1456	0.4052	0.6612	0.5782	0.2122	0.5026
PQA-Net [8]	0.5835	0.6470	0.6318	0.6358	0.7359	0.7857	0.5349	0.3762	0.8234	0.8653
IT-PCQA [20]	0.3570	0.7859	0.5913	0.4737	0.5467	0.5536	0.3777	0.4976	0.1790	0.4694
Proposed	0.8563	0.8686	0.7433	0.8433	0.7791	0.8315	0.8809	0.9257	0.8780	0.8955

**Table 9:** SRCC results of PCQA metrics on the WPC database across various distortion types. The top three results are highlighted in red (1st), blue (2nd), and green (3rd).

Types	Metric	Downsampling	Gaussian Noise	G-PCC (T)	V-PCC	G-PCC (O)
FR	${\mathrm{PSNR}}_{mse,p2po}$ [51]	0.4815	0.6155	0.3451	0.1602	NaN
	${\mathrm{PSNR}}_{mse,p2pl}$ [3]	0.3251	0.6194	0.3568	0.1992	NaN
	${\mathrm{PSNR}}_{hf,p2po}$ [51]	0.5356	0.6149	0.2811	0.2051	NaN
	${\mathrm{PSNR}}_{hf,p2pl}$ [3]	0.4879	0.6150	0.3085	0.2370	NaN
	${\mathrm{AS}}_{rms}$ [53]	0.2465	0.6844	0.1342	0.3877	0.0350
	${\mathrm{PSNR}}_{Y}$ [52]	0.5542	0.7644	0.5916	0.3203	0.8072
	PCQM [4]	0.4537	0.8775	0.7775	0.5534	0.8944
	PointSSIM [57]	0.8319	0.5844	0.6745	0.3546	0.7917
	GraphSIM [60]	0.7903	0.7469	0.7457	0.5989	0.8258
	SSIM [2]	0.8234	0.6264	0.4669	0.5141	0.5290
	MS-SSIM [37]	0.8834	0.7118	0.6042	0.5812	0.7214
	IW-SSIM [38]	0.8822	0.8560	0.6742	0.7063	0.7128
	VIFP [39]	0.8828	0.8847	0.6304	0.7410	0.7116
RR	PCMRR [7]	0.7407	0.7762	0.2702	0.2966	0.6468
NR	3D-NSS [12]	0.7508	0.7460	0.5947	0.3927	0.2891
	ResSCNN [11]	0.2899	0.5459	0.2531	0.1028	0.0247
	PQA-Net [8]	0.7234	0.7938	0.4710	0.0045	0.4204
	IT-PCQA [20]	0.3327	0.1718	0.1987	0.0090	0.1180
	Proposed	0.8738	0.8679	0.7826	0.7296	0.9073

**Table 10:** Ablation study (GATF) on ICIP2020, SJTU-PCQA, and WPC databases. For every feature combination, the table reports the result **without GATF** (w/o), **with GATF** (w/), and the relative **Gain** (%). The top three rankings for PLCC and SRCC are shown in red, blue, and green, for first, second, and third positions, respectively.

Dataset	Measure	Single Features			LAB + CURV			LAB + SAL			CURV + SAL			LAB + CURV + SAL		
		LAB	CURV	SAL	w/o	w/	Gain (%)	w/o	w/	Gain (%)	w/o	w/	Gain (%)	w/o	w/	Gain (%)
SJTU-PCQA	PLCC	0.76	0.78	0.86	0.77	0.82	**+6.25%**	0.81	0.83	**+3.24%**	0.82	0.95	**+15.94%**	0.80	0.90	**+12.50%**
	SRCC	0.77	0.77	0.85	0.77	0.82	**+6.73%**	0.81	0.85	**+5.26%**	0.81	0.93	**+14.81%**	0.80	0.90	**+13.14%**
WPC	PLCC	0.71	0.68	0.74	0.69	0.70	**+1.20%**	0.72	0.78	**+8.30%**	0.71	0.80	**+13.23%**	0.71	0.80	**+12.74%**
	SRCC	0.69	0.65	0.71	0.67	0.73	**+9.82%**	0.70	0.80	**+14.75%**	0.68	0.87	**+27.38%**	0.68	0.82	**+20.95%**
ICIP2020	PLCC	0.85	0.90	0.93	0.88	0.92	**+5.05%**	0.89	0.95	**+6.34%**	0.91	0.98	**+7.59%**	0.89	0.98	**+9.13%**
	SRCC	0.88	0.92	0.92	0.90	0.93	**+3.38%**	0.90	0.96	**+6.36%**	0.92	0.99	**+7.56%**	0.91	0.98	**+7.81%**

**Table 11:** Results of the cross-database evaluation. The notation “WPC→SJTU” indicates training on the WPC database and testing on the SJTU-PCQA database. Conversely, “SJTU→WPC” indicates training on the SJTU-PCQA database followed by testing on the WPC database. The best performance is highlighted in red.

Method	WPC→SJTU		SJTU→WPC	
	PLCC	SRCC	PLCC	SRCC
3D-NSS [12]	0.2344	0.1817	0.1422	0.1512
ResSCNN [11]	0.4089	0.4031	0.2690	0.2580
PQA-net [8]	0.6102	0.5411	0.2750	0.2650
IT-PCQA [20]	0.4710	0.4820	0.4290	0.4100
GPA-Net [66]	0.5740	0.5350	0.4310	0.4240
MM-PCQA [47]	0.7403	0.7278	0.3011	0.2101
GMS-3DQA [46]	0.7611	0.7421	0.3321	0.3196
Proposed method	0.7902	0.7615	0.5099	0.5144

## Data Availability

The datasets utilized in this study are publicly available and were sourced from the following repositories: the ICIP2020 dataset (http://emergimg.di.ubi.pt/icip2020PC.html, accessed on 5 January 2021), the SJTU-PCQA dataset (https://vision.nju.edu.cn/28/fd/c29466a469245/page.htm, accessed on 5 January 2021), and the WPC dataset (https://drive.google.com/drive/folders/1dHDqKXgvkUhQdUzT7pJjrJ7zRnceFIkO, accessed on 5 January 2021).

## Abbreviations

The following abbreviations are used in this manuscript:

PC	Point Cloud
PCQA	Point Cloud Quality Assessment
NR	No Reference
FR	Full Reference
RR	Reduced Reference
GATF	Graph Attention Transformer Fusion
GAT	Graph Attention Network
GCN	Graph Convolutional Network
MOS	Mean Opinion Score
PLCC	Pearson Linear Correlation Coefficient
SRCC	Spearman Rank Correlation Coefficient
KRCC	Kendall Rank Correlation Coefficient
RMSE	Root Mean Squared Error
RBF	Radial Basis Function
CNN	Convolutional Neural Network
DNN	Deep Neural Network
G-PCC	Geometry-based Point Cloud Compression
V-PCC	Video-based Point Cloud Compression
OT	Octree-based Compression
GGN	Gaussian Geometry Noise
CN	Color Noise
DS	Downsampling
GPU	Graphics Processing Unit
